# Inhibition of (pro)renin Receptor Contributes to Renoprotective Effects of Angiotensin II Type 1 Receptor Blockade in Diabetic Nephropathy

**DOI:** 10.3389/fphys.2017.00758

**Published:** 2017-10-06

**Authors:** Lin Zhang, Xiao-Fei An, Xin Ruan, Dong-Dong Huang, Li Zhou, Hong Xue, Li-Min Lu, Ming He

**Affiliations:** ^1^Department of Biochemistry and Molecular Cell Biology, Shanghai Jiao Tong University School of Medicine, Shanghai, China; ^2^Department of Endocrinology, Jiangsu Province Hospital of Chinese Medicine, Affiliated Hospital of Nanjing University of Chinese Medicine, Nanjing, China; ^3^Key Laboratory of Cell Differentiation and Apoptosis of Chinese Ministry of Education, Department of Pathophysiology, Shanghai Jiao Tong University School of Medicine, Shanghai, China; ^4^Department of Physiology and Pathophysiology, Fudan University Shanghai Medical College, Shanghai, China

**Keywords:** diabetic nephropathy, renin-angiotensin system, (pro)renin receptor, angiotensin II type 1 receptor, angiotensin II type 2 receptor

## Abstract

**Aims:** Renal renin-angiotensin system (RAS) plays a pivotal role in the development of diabetic nephropathy (DN). Angiotensin II (Ang II) type 1 receptor (AT_1_R) blockade elevates (pro)renin, which may bind to (pro)renin receptor (PRR) and exert receptor-mediated, angiotensin-independent profibrotic effects. We therefore investigated whether PRR activation leads to the limited anti-fibrotic effects of AT_1_R blockade on DN, and whether PRR inhibition might ameliorate progression of DN.

**Methods:** To address the issue, the expression of RAS components was tested in different stages of streptozotocin (STZ)-induced diabetic rats (6, 12, and 24 weeks) and 6-week AT_1_R blockade (losartan) treated diabetic rats. Using the blocker for PRR, the handle region peptide (HRP) of prorenin, the effects of PRR on high glucose or Ang II-induced proliferative and profibrotic actions were evaluated by measurement of cell proliferation, matrix metalloproteinase-2 (MMP-2) activity, activation of extracellular signal-regulated kinase 1/2 (ERK1/2) and transforming growth factor-β1 (TGF-β1) expression in rat mesangial cells (MCs).

**Results:** PRR was downregulated in the kidneys of different stages of diabetic rats (6, 12, and 24 weeks). Moreover, 6-week losartan treatment further suppressed PRR expression via upregulating AT_2_R, and ameliorated diabetic renal injury. HRP inhibited high glucose and Ang II-induced proliferative and profibrotic effects in MCs through suppressing TGF-β1 expression and activating MMP-2. Meanwhile, HRP enhanced losartan's anti-fibrotic effects through further inhibiting phosphorylation of ERK1/2 and TGF-β1 expression. Moreover, the inhibitive effect of HRP on Ang II-induced TGF-β1 expression depended on the regulation of PRR expression by AT_2_R.

**Conclusions:** Our findings suggest that inhibition of PRR contributes to renoprotection against diabetic nephropathy by AT_1_R blockade.

## Introduction

Diabetic nephropathy (DN) is one of the most important long-term complications of diabetes and the major cause of end-stage renal disease and mortality in diabetic patients (Reidy et al., 2014). Activated renal tissue-localized renin-angiotensin system (RAS) plays an important role in the development of kidney disease in diabetes (Rahimi, 2016). Though multiple clinical trials have shown that angiotensin-converting enzyme inhibitors (ACEI) (Lewis et al., 1993) and Angiotensin II (Ang II) type 1 receptor (AT_1_R) blockades (ARB) (Brenner et al., 2001) attenuate DN, these effects were limited and were unable to halt the progression of DN into end-stage organ failure (Zhang et al., 2011). One of the most remarkable changes during Ang II blockade treatment is the increased production of prorenin/renin in plasma and kidney (Gomez et al., 1990; Zhang et al., 2011). Increased plasma prorenin concentration is associated with microalbuminuria in patients with diabetes mellitus (Deinum et al., 1999). Moreover, in addition to produce Ang II, prorenin/renin had its receptor, (pro)renin receptor (PRR) (Nguyen et al., 2002). The binding of prorenin/renin to PRR exerts angiotensin-independent, receptor-mediated profibrotic effects through activating intracellular signal transductions such as extracellular-signal-regulated kinase (ERK) and p38MAPK (Nguyen et al., 2002). We hypothesize that PRR activation may limit the anti-fibrotic effects of angiotensin blockade in DN, and thus, PRR inhibition may enhance the therapeutic effects. In 2004, Ichihara et al. synthesized a decoy peptide, called “handle region peptide” (HRP), which serves as a PRR blocker (Ichihara et al., 2004). HRP prevented the development of glomerulosclerosis in human PRR transgenic rats (Kaneshiro et al., 2007). Our previous studies showed that both PRR siRNA and HRP inhibited mesangial cells (MCs) proliferation and reduced associated fibrotic factor release, including transforming growth factor-β1 (TGF-β1) and matrix metalloproteinase-2 (MMP-2) (He et al., 2009). However, the interaction between PRR and DN has not been well-studied.

In this study, we aim to investigate whether PRR plays a role during different stages of DN (from early to end stage) and during losartan (AT_1_R blockade) treatment, especially in the setting of high glucose and Ang II-related renal fibrosis *in vivo* and *in vitro*. Our findings demonstrate that the kidney PRR is downregulated in diabetic rats and is further significantly suppressed after 6 weeks of losartan treatment. HRP inhibits high glucose and Ang II-induced proliferative and profibrotic effects in MCs through suppressing ERK1/2 activation, TGF-β1 expression and activating MMP-2. Co-treatment of HRP and losartan produced an additive anti-fibrotic effects through further inhibiting phosphorylation of ERK1/2 and TGF-β1 expression. HRP blunts Ang II-induced TGF-β1 expression through regulating PRR via AT_2_R in MCs. Our findings highlight blockade of PRR as a possible new therapy for DN.

## Methods

### Animals and experimental protocol

All animal procedures were carried out in accordance with the guidelines for the Care and Use of Laboratory Animals of Shanghai Jiao Tong University School of Medicine and approved by the Institutional Animal Care and Use Committee (Department of Laboratory Animal Science, Shanghai Jiao Tong University School of Medicine). Male Sprague-Dawley (SD) rats (150–200 g) were supplied by Shanghai SLAC Laboratory Animal Co. LTD (Shanghai, China). All animals were housed under standard laboratory conditions (12 h light/12 h dark, temperature 22–26°C, air humidity 55–60%) with *ad libitum* water and rat chow. The STZ-induced diabetic rat models in different stages were constructed as previously described (Tesch and Allen, 2007; He et al., 2010). Plasma level of glucose was measured using blood glucose kit assays (Jiancheng Bioengineering Company, Nanjing, China) 1 week after STZ administration. Rats with plasma glucose higher than 16.7 mM were used in the present study. The blood glucose, urine volume, urine protein excretion, and serum creatinine were measured as described previously (He et al., 2010).

For losartan treatment experiments, STZ-induced diabetic rats were further divided randomly into three groups: one was treated with losartan (gift of Hangzhou MSD Pharmaceutical Co. Ltd., Zhejiang, China) at a dose of 20 mg/kg body weight per day by gavage once daily (*n* = 8) for 6 weeks (beginning 1 week until 7 weeks after STZ administration); another group DM rats (*n* = 8) was given equal volume of water by gavage administration for 6 weeks. The third group, i.e., the non-diabetic rats, was used as the Control group (*n* = 8) and was given equal volume of water via gavage administration for 6 weeks. All the rats in these three groups were anesthetized and sacrificed after 6 weeks of losartan treatment to obtain the blood sample and kidney of each animal.

### Histology and immunohistochemistry

Rat kidneys collected from different groups were immediately fixed in 4% formaldehyde, and were then embedded in paraffin. Paraffin-embedded kidney sections (5 μm) were analyzed after hematoxylin & eosin (H&E) staining and periodic acid-Schiff (PAS) staining.

For immunohistochemistry, after deparaffinization and hydration through xylenes, slides were subjected to microwave for antigen retrieval. Endogenous peroxidase activity was quenched and sections were incubated with rabbit serum for 20 min, followed by incubation overnight at 4°C with a 1:100 dilution of the primary antibody, rabbit anti- rat ATP6IP2/renin receptor antibody (Abcam, Cambridge, UK, 1:100 dilution). The PRR was then detected using a commercial immunoperoxidase staining kit (Boster ABC kit, Wuhan, China). Briefly, the sections were incubated with a 1:100 dilution of biotinylated secondary goat anti-rabbit antibody for about 30 min at 37°C, followed by avidin-biotin-peroxidase complex (ABC) reagent incubation for 30 min at 37°C. Bound antibody conjugates were visualized using 3,3′-diaminobenzidine (DAB) as a chromogen to develop a brown stain and mounted with glycerol gelatin. The sections were not counterstained with hematoxylin to better compare PRR expression (Deng et al., 2006).

### Electron microscopy analysis

Rat kidneys were fixed in 2.5% glutaraldehyde in sodium cacodylate buffer. Samples were post-fixed in OsO4, dehydrated in ethanol, and embedded in resin. Ultrathin sections (50~60 nm) were counterstained with uranyl acetate and lead citrate and examined with a Philips CM120 transmission electron microscope.

### Measurements of the components of RAS

For the determination of plasma renin activity, plasma AngII and kidney AngII, we treated the plasma and kidney as previously reported (He et al., 2009, 2010). Plasma renin activity was determined by the rate of angiotensin I (AngI) generation from angiotensinogen at a substrate concentration close to Km. Plasma renin activity was determined with Ang I radioimmunoassay kit (Beijing North Institute of biological technology, China) and plasma Ang II and kidney Ang II were determined with Ang II radioimmunoassay kit (Beijing North Institute of biological technology, China).

### RNA extraction and real-time PCR

Total RNA of isolated renal cortexes or MCs was extracted using Trizol reagent (Invitrogen, Carlsbad, CA, USA) according to the manufacturer's instructions. Complementary DNA was synthesized with the Prime-Script RT reagent kit (Promega, Madison, WI). Real-time PCR was performed using SYBR Green PCR Master Mix (Applied Biosystems, CA) on an ABI 7900HT fast real-time PCR system (Applied Biosystems). Expression data were normalized to internal GAPDH and the relative expression levels were evaluated using the ΔΔCt method (He et al., 2010, 2013; Zhang et al., 2016). Primer sequences used in real-time PCR are shown in Table S1. The levels of target gene mRNA were calculated as a percentage of those in control MC or kidney. The data were expressed as average of triplicates ± SEM. The same experiments were repeated 3–5 times.

### Western blotting analysis

Cell lysates were fractionated by sodium dodecyl sulfate (SDS)-polyacrylamide gelelectrophoresis and then transferred to nitrocellulose membrane (Axygen, Union City, CA). After blocking with 5% nonfat milk in Tris-buffered saline, the membranes were incubated with the antibodies against anti-ATP6IP2/renin receptor antibody (Abcam, Cambridge, UK, 1:1,000 dilution), anti-phosphorylation of ERK1/2 (Cell Signaling Technology, Beverly, MA, 1:1,000 dilution), anti-ERK1/2 (Cell Signaling Technology, Beverly, MA, 1:1,000 dilution) or anti-β-actin (Merck, Darmstadt, Germany, 1:5,000 dilution) followed by horseradish peroxidase (HRP)-linked secondary antibodies (KPL, Guildford, UK, 1:2,000 dilution) for 1 h at room temperature. The filter was finally washed three times and treated with enhanced chemiluminescent reagents, exposed to Kodak X-ray film for 1–20 min to detect the signals.

### Cell culture

The immortalized rat renal mesangial cell line was kindly provided by the Department of pathology of Fudan University, China (He et al., 2009). The cells were incubated in Dulbecco's modified Eagle medium (DMEM, Sigma-Aldrich, St. Louis, MO, USA) containing normal glucose concentration of 5.56 mM D-glucose and supplemented with 10% new bovine serum (NBS) in a humidified atmosphere of 95% air and 5% CO_2_ at 37°C. To observe the effect of high glucose, D-glucose (Sigma-Aldrich) was added into the culture medium to increase the glucose concentration to 30 mM; and 24.44 mM mannitol (Sigma-Aldrich, St. Louis, MO, USA) was added instead of glucose to serve as osmolality control.

### Fluorescence microscopy studies on binding of HRP and PRR

To study the binding of HRP and PRR in MCs, we synthesized HRP labeled by FITC at the C terminal of the decapeptide, and its sequence is FITC-RILLKKMPSV-COOH (He et al., 2009). Treated with FITC-HRP for 5 min, MCs were washed by cold PBC twice, fixed, blocked, incubated with the anti-ATP6IP2/renin receptor antibody (Abcam, Cambridge, UK, 1:100 dilution), followed by 1 h of incubation at room temperature with Rhodamine (TRIC) labeled anti-goat antibody (rabbit polyclonal; 1:50; KPL, USA) in blocking buffer. Images were then obtained via the laser scanning confocal microscope (Leica, Germany). The co-localization was detected in a triple fluorescence photograph using View-sect software (Leica, Germany).

### MMP-2 activity determination by gelatin zymography

The MMP-2 enzyme activity was determined by gelatin zymography as described previously (He et al., 2009). The ratio of the densities of active MMP-2 (68 kD) to total MMP-2 (the sum of 68 and 72 kD) represents the activity of MMP-2 in the culture medium. The experiment was performed in triplicate.

### Cell proliferation assay

Cell proliferation of MCs was measured using a colorimetric 5-bromo-2′-deoxyuridine (BrdU) enzyme-linked immunosorbent assay (ELISA) (Roche Applied Science, USA.) according to the manufacturer's protocol. The developed color of the reaction product and thereby the absorbance values difference of 370 and 492 nm (A370–A492 nm) correlates directly to the amount of DNA synthesis, and thus, cell proliferation. The experiments were performed in triplicate.

### Statistical analysis

All values are presented as mean ± SEM. The statistical significance of differences among groups was assessed by using one way-ANOVA, followed by Student-Newman-Keul test. A *P*-value of < 0.05 was taken as statistically significant.

## Results

### Renal damage in the different stages of STZ-induced diabetic duration

**Figure 1:**
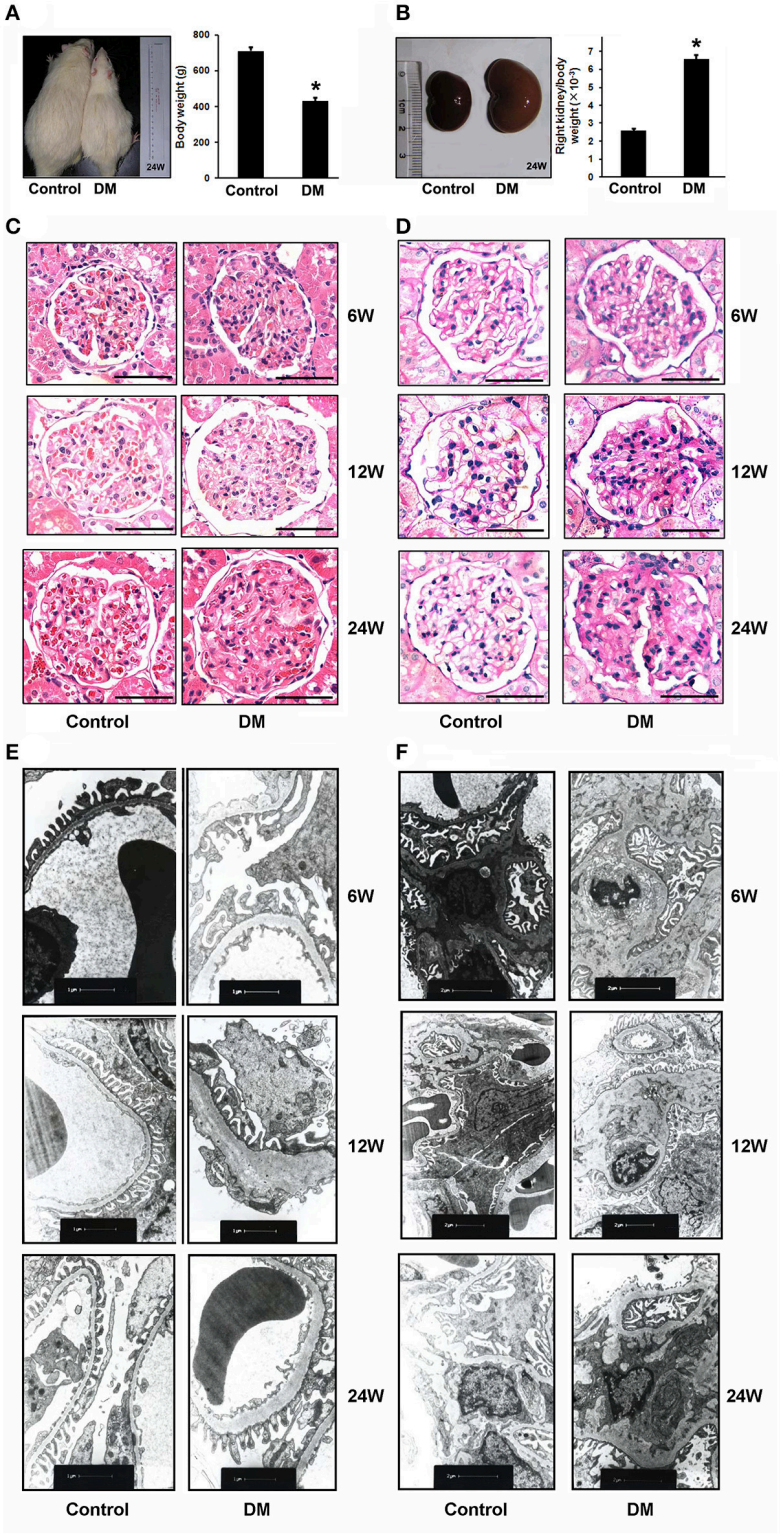
Evidence for diabetic nephropathy (DN) in different stages of STZ-induced diabetic rats. **(A)** Body weight, **(B)** ratio of right kidney to body weight of the Control (*n* = 7) and diabetic rats (DM rats) (*n* = 6) in 24 weeks after STZ administration. The photographs are the representative for rats and kidneys. **(C)** Representative hematoxylin and eosin (H&E) staining and **(D)** periodic acid-Schiff (PAS) staining of glomeruli from Control and DM rats in the different diabetes stages (6, 12, and 24 weeks). Scale bar: 50 μm. **(E,F)** Electron microscopy of kidney sections from Control and DM rats in the different diabetes stages (6, 12, and 24 weeks after STZ administration). **(E)** Representative electron microscopy images of glomerular basement membranes and podocytes in glomeruli. Scale bar: 1 μm. **(F)** Representative electron microscopy images of mesangial areas in glomeruli. Scale bar: 2 μm. ^*^P < 0.05 Control vs. DM rats.

To investigate the role of PRR in DN, we constructed a STZ-induced diabetic rat model and the rats with blood glucose higher than 16.7 mM were included in this study. In the different stages of STZ-induced diabetic duration (6, 12, and 24 weeks after STZ administration), the urine protein excretion and metabolic indices, including blood glucose, body weight, and urine output of each group were collected (Table 1). After 6 or 12 weeks, the survival rates of both Control and diabetic mellitus (DM) group were 100% (7/7). But the survival rate of DM rats in 24 weeks was decreased to 75% (6/8), while no rats in Control group (7/7) died (Table 1). All of the DM rats in the three stages exhibited progressive increase in blood glucose level, urinary protein excretion, and kidney weight, but showed a decrease in body weight (Figures 1A–C and Table 1). Moreover, the urinary protein excretion in DM rats increased with time (2.74, 6.03, and 10.95-fold higher in DM than age-matched Control group at 6-, 12-, and 24-weeks). Renal pathological examination by H&E and PAS staining indicated glomerular hypertrophy, expansion of mesangium, and increased diffusely appearing deposits in the basement membranes of capillary loops of the glomeruli in DM rats (Figures 1C,D). Electron microscopy showed glomerular basement membrane thickening (Figure 1E), increase of mesangial matrix (Figure 1F), and extensive podocyte fusion in model groups (Figure 1E). The severity of renal morphological changes increased with time, approximating diffuse diabetic glomerulosclerosis at advanced DN stages. This series study provided a successful model for investigating the role of PRR in DN development.

**Table 1:** Blood glucose, kidney weight, urine volume, and urinary protein excretion in control rats (Control) and STZ-induced diabetic rats (DM).

	**6W**		**12W**		**24W**	
	**Control (*n* = 7)**	**DM (*n* = 7)**	**Control (*n* = 7)**	**DM (*n* = 7)**	**Control (*n* = 7)**	**DM (*n* = 6)**
Blood glucose (mmol/l)	7.2 ± 0.2	37.3 ± 1.9^*^	6.8 ± 0.2	30.3 ± 1.4^*^	6.9 ± 0.2	37.3 ± 1.1^*^
Body weight (g)	416.4 ± 9.1	345.6 ± 10.5^*^	548.9 ± 12.1	399.5 ± 14.3^*^	711.1 ± 22.2	430.8 ± 18.5^*^
Right kidney/body weight (×10^−3^)	3.3 ± 0.1	5.8 ± 0.4^*^	2.9 ± 0.1	5.9 ± 0.3^*^	2.6 ± 0.1	6.6 ± 0.2^*^
Urine volume (ml/d)	21.2 ± 3.6	208.4 ± 13.6^*^	27.3 ± 3.4	275.3 ± 20.0^*^	30.3 ± 2.7	337.2 ± 22.1^*^
Urine protein excretion (mg/d)	32.6 ± 2.6	89.3 ± 6.2^*^	17.8 ± 1.3	107.3 ± 6.3^*^	17.4 ± 1.3	190.6 ± 13^*^

Data are expressed as mean ± SEM.

* *P < 0.05 compared with control rats in same time point*.

### Expression of PRR and the other components of RAS in the kidney and plasma of diabetic rat

**Figure 2:**
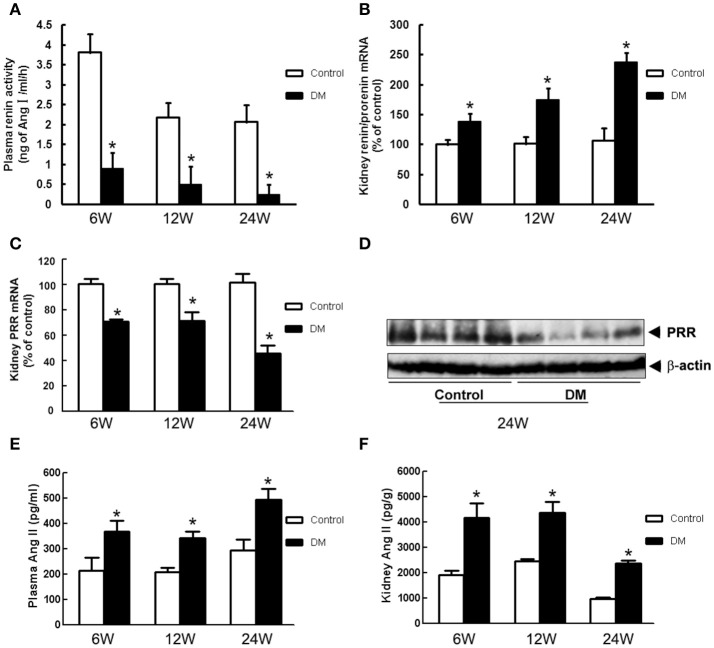
RAS components in the plasma and kidney of Control and DM rats at the end of 6, 12, or 24 weeks after STZ induction. **(A)** Plasma renin activity measured by radioimmunoassay. **(B)** Kidney (pro)renin and **(C)** PRR mRNA level by real-time PCR. **(D)** Kidney PRR protein level by Western blotting. **(E)** Plasma and **(F)** kidney Ang II measured by radioimmunoassay. ^*^P < 0.05 Control vs. DM rats in the same time point.

We tested the components of RAS in kidneys of all groups. Plasma renin activity was significantly lower in DM rats than in the age-matched Control rats from early to end stage DN (at stages of 6-, 12-, and 24-week diabetic duration) (Figure 2A), but kidney (pro)renin mRNA was higher in DM rats (Figure 2B). Both PRR mRNA and protein levels in the renal cortex of DM rats were significantly lower than those of the Control group (Figures 2C,D). Meanwhile, DM rats showed a significant increase in plasma and kidney Ang II (Figures 2E,F).

### Renal-protective effects of AT_1_R blockade (losartan) in diabetic rats

AT_1_R blockade losartan treatment (20 mg/kg body weight per day by gavage for 6 weeks) attenuated, but did not completely reverse hyperglycaemia, renal hypertrophy, polyuria, proteinuria and elevations of serum creatinine of DM rats (Table 2). Losartan had no effect on the body weight of DM rats. H&E (Figure 3A), PAS staining (Figure 3B), and electron microscopy (Figures 3C,D) showed that the changes of DN in 6 weeks, including glomerular hypertrophy, expansion of mesangium, mesangial matrix increase, glomerular basement membrane thickening and extensive podocyte fusion, were attenuated by losartan treatment. These data suggest that losartan exhibits a renoprotective effect in STZ-induced rats, but cannot completely halt the progression of DN into end-stage organ failure.

**Table 2:** Effects of 6-week losartan treatment on blood glucose, kidney weight, urine protein excretion, and serum creatinine of STZ-induced diabetic rats.

**Group**	***n***	**Blood glucose (mmol/l)**	**Body weight (g)**	**Right kidney/body weight (×10^−3^)**	**Urine volume (ml/d)**	**Urine protein excretion (mg/d)**	**Serum creatintine (μmol/l)**
Control	8	6.9 ± 0.2	465.1 ± 11.3	3.1 ± 0.1	20.6 ± 3.1	22.5 ± 2.6	61.5 ± 7.4
DM	8	25.3 ± 1.3^*^	348.1 ± 11.3^*^	6.7 ± 0.1^*^	208.3 ± 13.2^*^	64.1 ± 7.3^*^	85.4 ± 5.8^*^
DM+losartan	8	19.6 ± 2.8^*^^#^	362.3 ± 23.1^*^	5.7 ± 0.7^*^	145.8 ± 37.8^*^^#^	37.8 ± 7.5^#^	67.7 ± 5.2^#^

Data are expressed as mean ± SEM.

* P < 0.05 compared with control rats;

# *P < 0.05 for DM+losartan rats compared with DM rats*.

**Figure 3:**
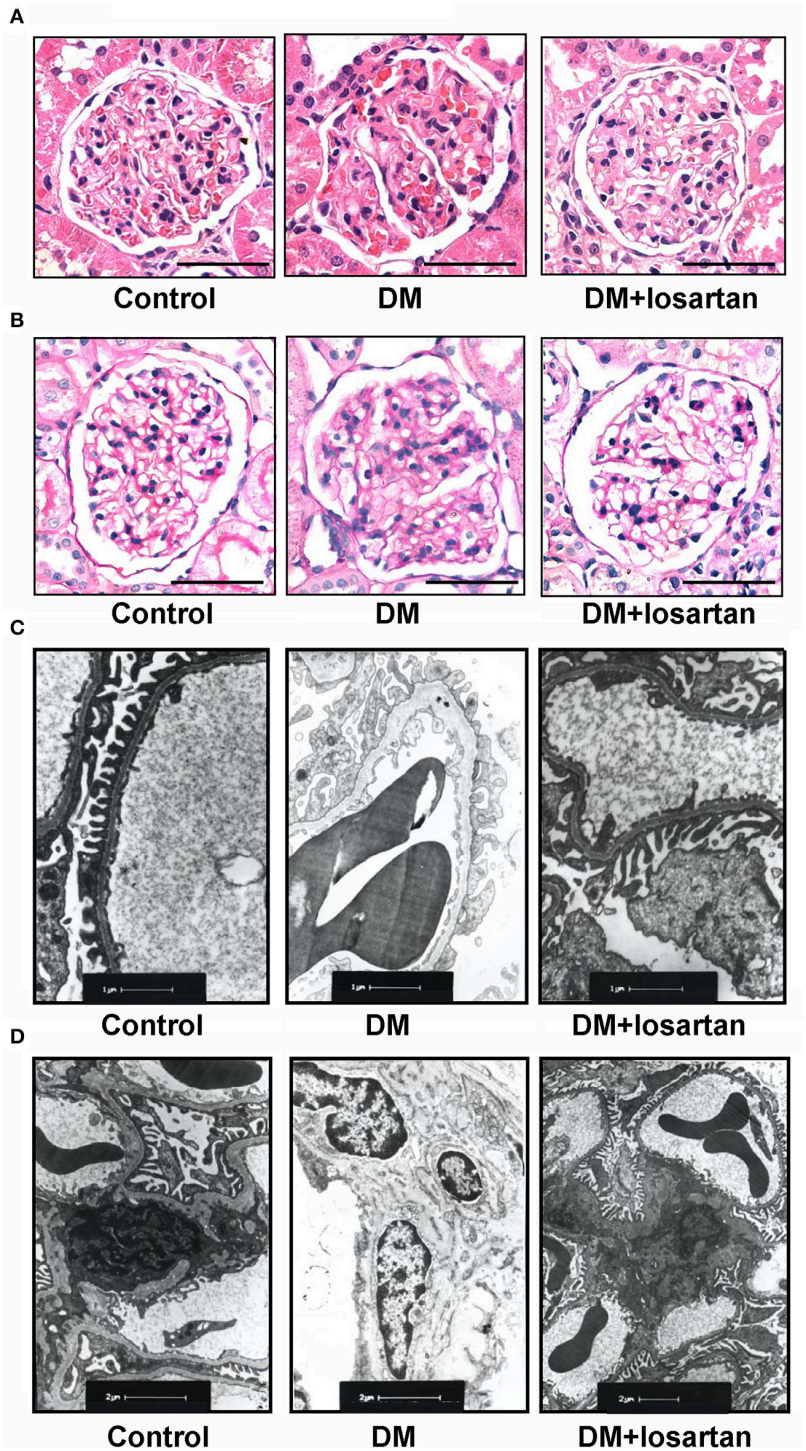
Histology analysis of kidneys from Control, DM and losartan treated DM (DM + losartan) rats. **(A)** Representative H&E staining and **(B)** PAS staining of glomeruli from Control, DM and DM + losartan rats. Scale bar: 50 μm. **(C,D)** Electron microscopy of kidney sections. **(C)** Representative electron microscopy images of glomerular basement membranes and podocytes in glomeruli. Scale bar: 1 μm. **(D)** Representative electron microscopy images of mesangial areas of glomeruli. Scale bar: 2 μm.

### Effects of losartan on the expression of PRR and TGF-β1 expression in kidneys of diabetic rats

**Figure 4:**
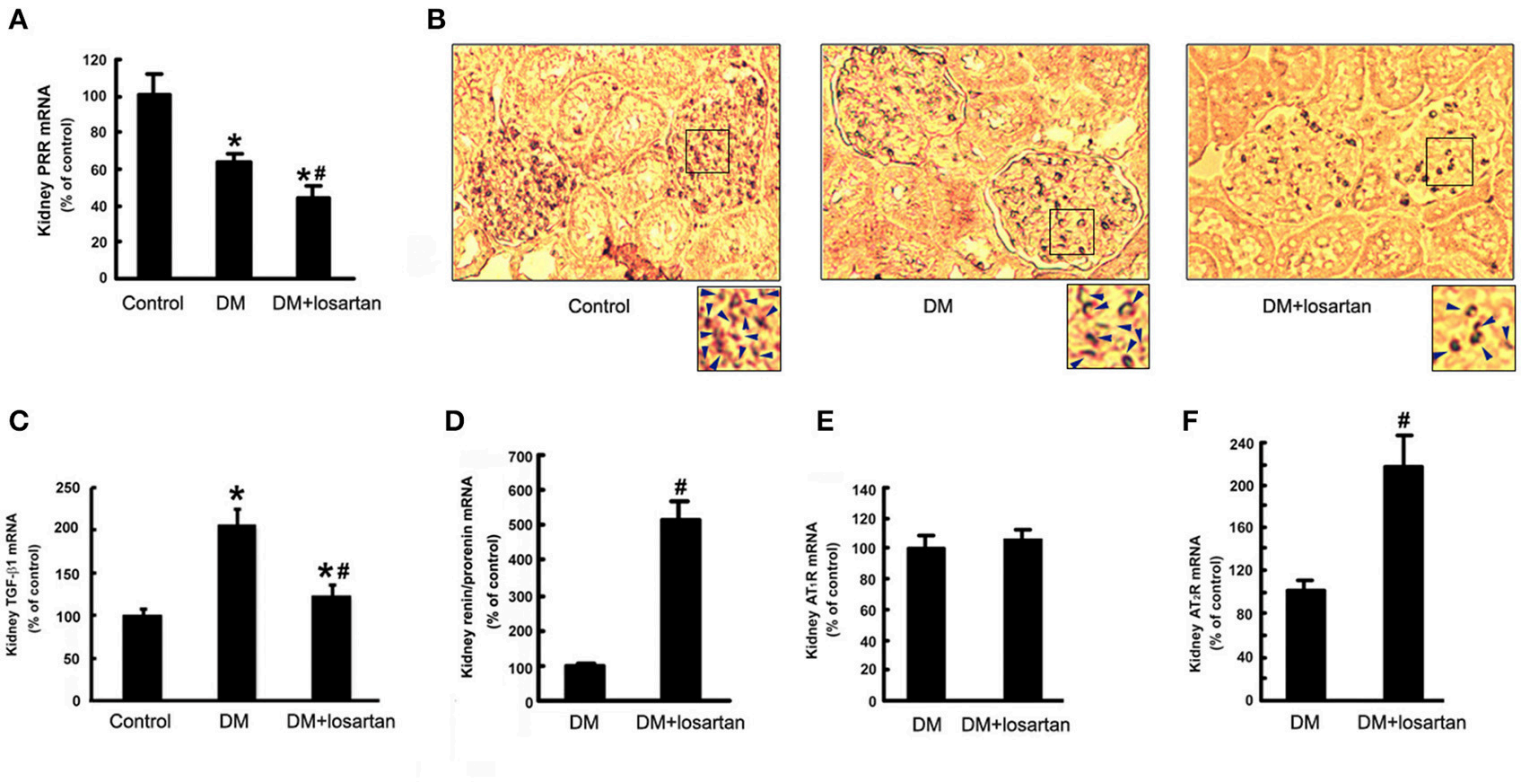
Expression of the components of RAS and TGF-β1 in the kidney of Control, DM and DM + losartan rats. **(A)** Kidney PRR mRNA level by real-time PCR. **(B)** Immunohistochemical staining for PRR in the kidneys of Control (*n* = 8), DM (*n* = 8) and DM + losartan (*n* = 8) rats (not counterstained with hematoxylin to compare the PRR expression better). The arrows indicated the representative area of PRR expression in the renal tissues. **(C)** TGF-β1, **(D)** (pro)renin, **(E)** AT_1_R, **(F)** AT_2_R mRNA level in the kidneys of DM (*n* = 8) and DM + losartan (*n* = 8) rats by real-time PCR. ^*^P < 0.05 compared with Control rats, ^#^P < 0.05 compared with DM rats.

To investigate the roles of PRR in the protective effects of losartan in DN, we tested the renal levels of PRR, TGF-β1, and other components of RAS in the Control, DM and DM treated with losartan group (DM + losartan). DM rats exhibited a reduction of kidney PRR mRNA. Notably, 6-week DM + losartan rats had lower PRR mRNA expression compared with DM rats (Figure 4A). Immunohistochemical staining showed that PRR mainly localized in the glomeruli mesangium zone (Figure 4B). Moreover, the kidney positive staining for PRR of the DM + losartan group was significantly lower than that of DM rats, similar to the real-time PCR results (Figure 4A). The increased kidney TGF-β1 mRNA of DM rats was attenuated by losartan, but the level was still higher than the Control group (Figure 4C). Meanwhile, the (pro)renin and AT_2_R mRNA levels of DM rats were significantly increased after losartan treatment (Figures 4D,F). However, losartan had no effect on the kidney AT_1_R level of DM rats (Figure 4E). These data suggest that AT_1_R blockade may play renoprotective roles not only by blocking the AT_1_R, but also by decreasing PRR.

### Co-localization of HRP and PRR

**Figure 5:**
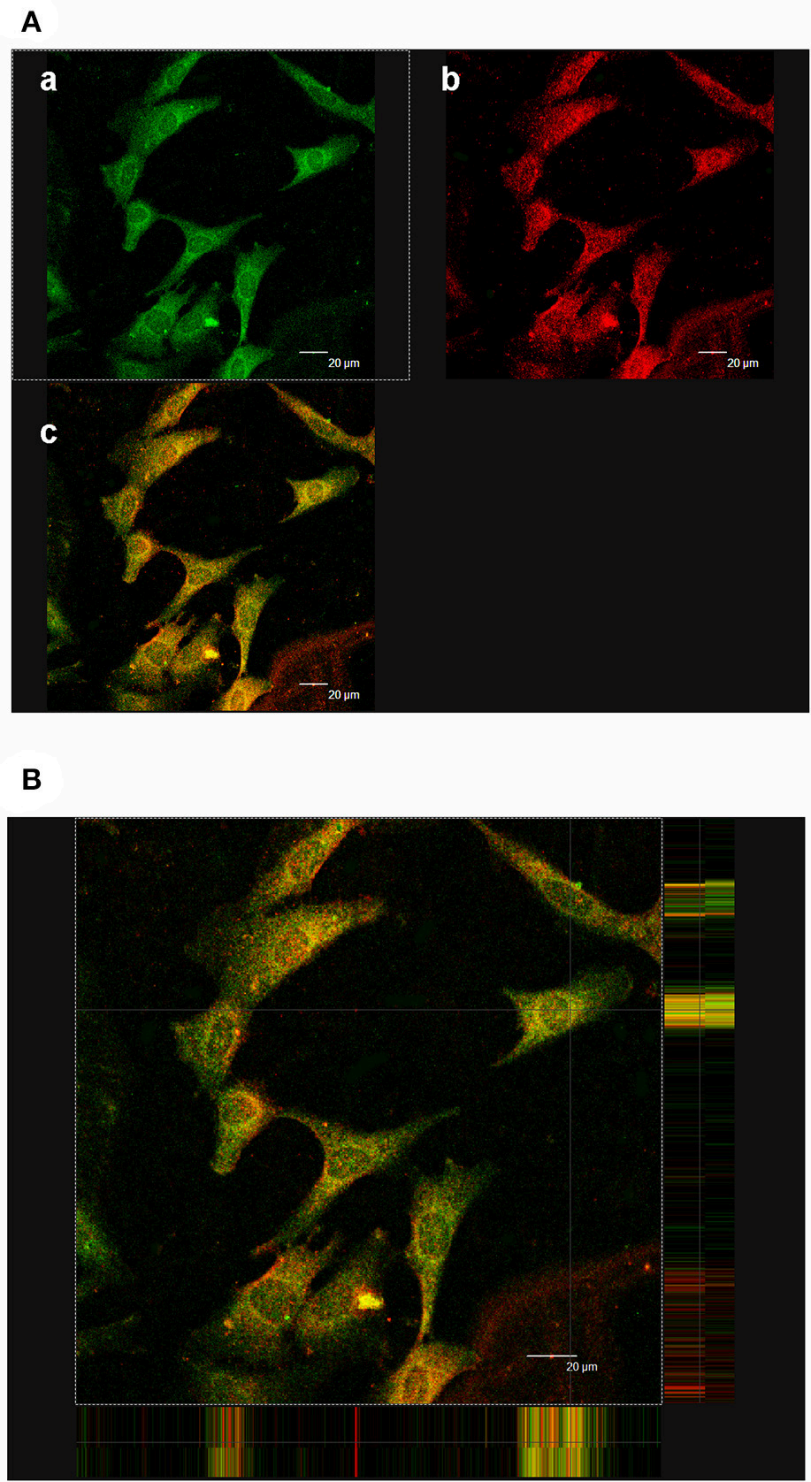
Co-localization of PRR and FITC-HRP. **(Aa)** Immunofluorescence showing FITC-HRP was mainly located in the cytoplasm and membrane. **(b)** Immunofluorescence showing PRR was mainly located in the cytoplasm and membrane. **(c)** Analysis of the overlay of PRR and HRP. Yellow is the co-localization. **(B)** Triple fluorescence photograph with confocal microscopy reveals the co-localization of PRR and FITC-HRP (5 min). The co-localization (yellow staining) mainly focused in the membrane and cytoplasm. Scale bar: 20 μm.

Our previous study reported the existence of (pro)renin mRNA in MCs and renin activity in the cultured medium (He et al., 2009). HRP (10^−6^ M), the blocker of PRR, was used to identify the roles of PRR in the MCs *in vitro*. *In vivo*, PRR was observed mainly in the mesangium of cortical glomeruli (Figure 4B). In MCs, PRR mainly localized to the perinuclear zone and plasma membrane using immunofluorescence (Figure 5Aa). FITC-labeled HRP (FITC-HRP) was synthesized to observe the cellular distribution of the HRP-PRR complex. FITC-HRP translocated from culture medium into the cytoplasm within 30 s. Triple fluorescence photograph by confocal microscopy showed that the co-localization of PRR and FITC-HRP was mainly on the cell membrane and in the perinuclear zone of the cytoplasm in 5 min after FITC-HRP treatment (Figures 5A,B). These data further suggest that HRP may interact with PRR in MCs.

### HRP suppresses high glucose-induced phosphorylation of ERK1/2, TGF-β1 expression and MMP-2 deactivation in MCs

**Figure 6:**
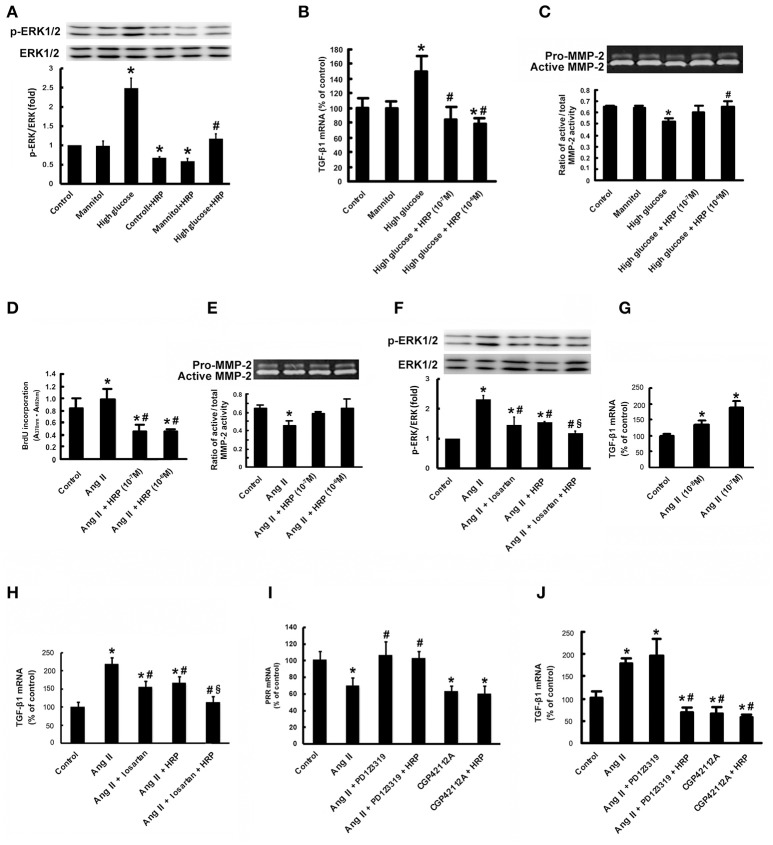
HRP suppressed high glucose and Ang II-induced proliferative and fibrotic effects in MCs. Antagonistic action of HRP (10^−7^ M and 10^−6^ M) on high glucose-induced increase in phosphorylation of ERK1/2 **(A)** and TGF-β1 mRNA expression **(B)** by real-time PCR. **(C)** Abolition of the high glucose-induced decrease in MMP-2 activity by HRP by gelatin zymography. ^*^P < 0.05 compared with control; ^#^*P* < 0.05 compared with high glucose group. **(D)** BrdU ELISA shows suppression of the Ang II-induced increase in proliferation of MCs by HRP (10^−7^ M and 10^−6^ M). **(E)** Abolition of the Ang II-induced decrease in MMP-2 activity by HRP (10^−7^ M and 10^−6^ M) by gelatin zymography. Co-treatment of HRP and losartan leads to a further reduction in phosphorylation of ERK1/2 **(F)** and TGF-β1 expressions **(H)** compared with losartan or HRP individual treatment. **(G)** Ang II causes a dose-dependent increase in TGF-β1 mRNA expression. **(I)** The suppressing effect of Ang II on PRR expression is induced by activation of AT_2_R. HRP has no effect on the PRR expression. **(J)** Effects of AT_2_R activation and PRR inhibition by HRP on TGF-β1 expressions in MCs by real-time PCR. ^*^P < 0.05 compared with control; ^#^*P* < 0.05 compared with Ang II group; ^§^*P* < 0.05 compared with individual treatment by losartan or HRP.

Since it has been reported that PRR activation promotes the production of profibrotic factors through activating ERK signaling, we examined the effect of HRP on high glucose-induced phosphorylation of ERK1/2 and TGF-β1 expression, one of the most important fibrotic factors in DN (Nguyen et al., 2002). Treatment with high glucose caused a significant increase in phosphorylation of ERK1/2 (Figure 6A) and TGF-β1 mRNA expression (Figure 6B), while no significant change was observed in MCs treated with mannitol (as osmolarity control). HRP (10^−6^ M) attenuated high glucose-induced phosphorylation of ERK1/2 to control group level (Figure 6A). HRP (10^−7^ and 10^−6^ M) reversed the increased TGF-β1 mRNA expression to control level or even lower level than control (Figure 6B). High glucose also induced a decrease in MMP-2 activity of the MCs, while HRP (10^−7^ M and 10^−6^ M) reversed the decreased MMP-2 activity to control or mannitol group levels (Figure 6C). These data suggest that PRR is activated and involved in high glucose-induced proliferative and profibrotic effects and HRP may eliminate these effects through blocking the activation of PRR in MCs.

### HRP suppresses Ang II-induced cell proliferation and MMP-2 deactivation in MCs

Besides high glucose, Ang II is another pivotal factor in the pathogenesis of DN *in vivo* as shown in Figure 2. HRP (10^−7^ M and 10^−6^ M) eliminated high Ang II-induced cell proliferation as shown by BrdU incorporation (Figure 6D). In addition, the reduction of MMP-2 activity induced by Ang II (10^−7^ M) at 72 h was attenuated by HRP (Figure 6E). These data show HRP blunts Ang II-induced proliferative and profibrotic actions in MCs, suggesting that PRR may be involved in the Ang II signaling pathway.

### Combined treatment with HRP and losartan has an additive effect on inhibiting phosphorylation of ERK1/2 and TGF-β1 expression in MCs

Ang II caused an increase in phosphorylation of ERK1/2 and a dose-dependent increase in TGF-β1 mRNA expression (Figures 6F,G). Losartan (10^−6^ M) or HRP (10^−6^ M) attenuated Ang II-induced phosphorylation of ERK1/2 and expression of TGF-β1 mRNA (Figures 6F,H). The level of phosphorylation of ERK1/2 and TGF-β1 expression in HRP or losartan individual treatment group is still higher than control group (Figures 6F,H). Notably, combined treatment with losartan and HRP led to a further reduction in phosphorylation of ERK1/2 (Figure 6F) and TGF-β1 expression (Figure 6H) to control level, suggesting that HRP may enhance losartan's anti-fibrotic effects on DN through further or fully inhibiting PRR.

### Inhibitive effects of HRP on the Ang II signaling pathway depends on PRR expression regulated by AT_2_R

As described in our previous studies (He et al., 2010), Ang II (10^−7^ M) or AT_2_R agonist (CGP42112A) (10^−6^ M) decreased the expression of PRR mRNA, while AT_2_R antagonist (PD123319) (10^−5^ M) abolished the Ang II-induced repression of PRR expression (Figure 6I). HRP (10^−6^ M) did not affect the downregulation of PRR by AT_2_R activation (Figure 6I). Ang II (10^−7^ M) stimulated TGF-β1 mRNA expression and CGP42112A (10^−6^ M) significantly reduced TGF-β1 mRNA expression after 24 h treatment (Figure 6J). HRP (10^−6^ M) suppressed the induction of TGF-β1 mRNA expression by combined treatment with Ang II (10^−7^ M) and PD123319 (10^−5^ M), but did not further reduce the expression of TGF-β1 induced by CGP42112A (10^−6^ M) (Figure 6J). These data indicate that Ang II-induced proliferative and profibrotic actions are mediated at least in part through PRR. Moreover, HRP inhibition on the Ang II signaling pathway is dependent on regulation of PRR expression by AT_2_R.

## Discussion

A pivotal role for the RAS in the pathogenesis and development of DN is widely accepted, based largely on work showing the attenuation of DN by ACEI (Lewis et al., 1993; Zhang et al., 2011) and ARBs (Brenner et al., 2001; Zain and Awan, 2014). However, these agents cannot halt the progression of DN into end-stage organ failure, possibly because of insufficient suppression of the intrarenal RAS, especially the increased production of prorenin (Gomez et al., 1990). Prorenin, the inactive proenzyme form of renin, may contribute to kidney damage by binding to and activating PRR (Nguyen et al., 2002). On binding to PRR, pro(renin) induces direct (receptor-mediated, angiotensin-independent) proliferative and profibrotic effects by increasing phosphorylation of ERK1/2, and increasing TGF-β1 and MMP-2 expression (Nguyen et al., 2002; He et al., 2009). Therefore, PRR activation may limit therapeutic effects of ACEI and ARB on DN, and thus, a PRR blocker may enhance their anti-fibrotic effects.

In this study, DN models of different duration (6, 12, and 24 weeks) were constructed and metabolic indices, renal function, and kidney histological morphology suggested that the severity of renal damage increased with duration of STZ treatment. This series study provides a means for investigating the role of RAS in DN development. Plasma renin activity was suppressed and kidney (pro)renin mRNA expression was upregulated in DM rats, reflecting suppressed systemic RAS activity, possibly resulting from increased kidney-specific RAS activity (Price et al., 1999; Oshima et al., 2014). The expression of PRR mRNA and protein in the kidneys of DM rats was significantly downregulated in all three stages. These results are in agreement with our previous study showing the downregulation of PRR expression in the kidneys of early stage diabetic rats (1 and 3 weeks after STZ injection) (He et al., 2010). The regulation of kidney PRR expression during DM has not been consistently shown. Huang et al showed that the expression of rat kidney PRR was significantly increased 6 weeks after development of diabetes (Siragy and Huang, 2008). However, other reports showed no difference between the kidney PRR expression levels of nondiabetic and diabetic subjects (Konoshita et al., 2006). This difference may be caused by (1) different high glucose, Ang II and (pro)renin levels *in vivo*: we tested these parameters as shown in Table 1 and Figure 2, but these were not shown in other reports; (2) mRNA and protein extracted from different regions of the renal tissues: our immunohistochemical staining showed PRR localized mainly to the glomeruli mesangium zone (Figure 4B), but other reports used whole kidney. The regulation of PRR in the different types of renal cells may be different.

The major interest of this study was focused on the roles of PRR in the renal-protective effects of AT_1_R blockade on DN. We showed that losartan exhibited a protective effect on DN, suppressing phosphorylation of ERK1/2 and TGF-β1 expression, which also occurred in the previous observations in animals and patients (Siragy and Huang, 2008; Huang et al., 2011; Anbar et al., 2016). PRR activation by binding (pro)renin may activate ERK1/2 signaling, resulting in the production of profibrotic factors in diabetic nephropathy, such as TGF-β1 (Nguyen et al., 2002). Even PRR is suppressed (Figure 6I), HRP blunts high glucose and Ang II-induced phosphorylation of ERK1/2 (Figures 6A,F) and TGF-β1 expression (Figures 6B,H) through blocking the PRR. These suggest that PRR is activated and involved in diabetic nephropathy development even PRR is downregulated by high glucose and Ang II. Therefore, a clinically relevant finding in the present study is that losartan may further downregulate PRR. This maybe one of the mechanisms through which losartan exerts its renoprotective effects in DN besides blocking AT_1_R. *In vivo*, losartan had a mild protective effect against diabetic glycemia (Table 2), which is similar to the findings of Murali and Goyal (Murali and Goyal, 2001). Meanwhile, kidney (pro)renin and AT_2_R levels, but not AT_1_R level, were significantly upregulated by losartan. So, increased tissue Ang II (Figure 2F) suppresses PRR expression via AT_2_R activation (Figure 6I), which is consistent with our previous studies (He et al., 2010). On the other hand, Schefe et al. reported that on the activation of PRR by (pro)renin, the direct interaction partner of PRR, promyelocytic leukemia zinc finger protein (PLZF) is translocated to the nucleus and repressed the transcription of the PRR itself (Schefe et al., 2006). The present results are in agreement with these findings: losartan-induced high (pro)renin levels suppressed PRR expression through a short negative-feedback loop, preventing excessive receptor activation. In addition, there is also evidence indicating that Ang II stimulation induces cytosolic PLZF to bind to AT_2_R at the plasma membrane, driving PLZF to translocate into the nucleus in cardiac hypertrophy (Senbonmatsu et al., 2003). We observed the expression of PLZF mRNA in both the MCs and rat kidney (data not shown). Therefore, the possibility of the involvement of PLZF in regulation of PRR by AT_2_R cannot be ruled out and we will further study the detailed mechanism.

**Figure 7:**
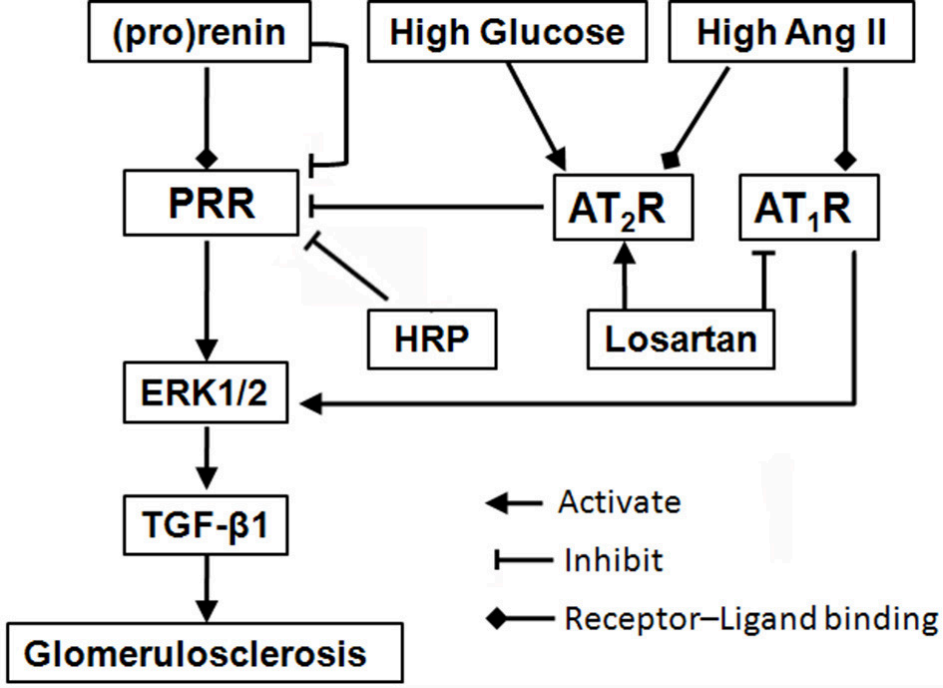
Schematic diagram of a proposed mechanism for blockade of (pro)renin/PRR activation as a therapeutic strategy. Binding of (pro)renin and PRR activates ERK1/2 signaling, resulting in the production of profibrotic factors in diabetic nephropathy, such as TGF-β1. During diabetes development, high glucose and Ang II, as well as high level (pro)renin, lead to a downregulation of PRR expression, through the mediation of AT_2_R (He et al., 2010) or PLZF (Schefe et al., 2006), indicating a negative feedback mechanism. But, the activation of the downregulated PRR still involes in the diabetic nephropathy development. Besides blocking AT_1_R, losartan plays renoprotective roles through further suppressing PRR via upregulation of AT_2_R. Moreover, HRP may enhance losartan's anti-fibrotic effects through further or fully inhibiting PRR. The present findings highlight blockade of PRR as a possible new addition therapy to AT_1_R blockade for DN. Abbreviations: Ang II, angiotensin II; AT_1_R, angiotensin II type 1 receptor; AT_2_R, angiotensin II type 2 receptor; ERK1/2, extracellular signal-regulated kinases 1 and 2; HRP, handle region peptide (HRP) of prorenin; PLZF, promyelocytic zinc finger; PRR, (pro)renin receptor; TGF-β1, transforming growth factor-β1.

On the basis of the available knowledge of PRR and present data, we hypothesize that in diabetic patients or animals, a high level of prorenin promotes renal MCs proliferation and fibrosis through binding to the PRR, which are further augmented by both high glucose and Ang II (via AT_1_R). On the other hand, prorenin, as well as high glucose and Ang II lead to a downregulation of PRR expression, possibly through PLZF (Schefe et al., 2006) and AT_2_R (Senbonmatsu et al., 2003; He et al., 2010), indicating a negative feedback mechanism (Figure 7). The final regulation and effects of PRR are determined by the balance of all of these factors. The activation of the downregulated PRR still involes in the diabetic nephropathy development. Besides blocking AT_1_R, losartan exerts renoprotection through further downregulating PRR via upregulation of AT_2_R (Figure 7). The present data suggest that downregulation of PRR plays an important adaptive and protective role in decreasing high glucose or Ang II induced renal fibrosis during diabetic condition.

HRP mimics the handle region of prorenin and binds competitively to PRR, thereby preventing receptor-mediated prorenin activation and intracellular signaling activation (Suzuki et al., 2003; Ichihara et al., 2004). In the present study, HRP eliminated the high glucose-induced profibrotic actions in MCs. Meanwhile, we firstly reported that HRP inhibited Ang II-induced MCs proliferation and MMP-2 deactivation, suggesting PRR is involved in Ang II-induced proliferative and profibrotic effects. The actions of Ang II are mainly via the signaling of AT_1_ and AT_2_ receptors (Senbonmatsu et al., 2003; Ding et al., 2016). Firstly, co-treatment of HRP and AT_1_R blockade (losartan) showed an additive inhibitory effect on Ang II-induced phosphorylation of ERK1/2 and TGF-β1 expression (Figure 6F). Since losartan totally blocked the AT_1_R, the effect of HRP cannot be explained on the basis of the suppression of local angiotensin generation, suggesting that HRP exerts beneficial effects in an Ang II-independent manner. This is consistent with the previous work showing that HRP inhibits the development of glomerulosclerosis in diabetic AT_1A_-receptor-deficient mice (Ichihara et al., 2006). Therefore, therapeutically induced increases in (pro)renin play a role in the limited effectiveness of Ang II blockade, and PRR blockade maybe a good addition to AT_1_R blockade for DN, especially for patients with high Ang II level (Figure 7). Secondly, HRP had no effect on the downregulationof PRR by activation of AT_2_R. HRP suppressed Ang II and AT_2_R blockade (PD123319)-induced increase of TGF-β1, but did not further reduce the decreased TGF-β1 expression by AT_2_R agonist (CGP42112A). This result indicated that the inhbitive effects of HRP on Ang II-induced cell proliferation and fibrosis depended on the regulation of PRR by AT_2_R.

In summary, the present study suggests inhibition of PRR may play a protective role in decreasing high glucose or Ang II-induced renal fibrosis during diabetic condition (Figure 7). We demonstrated that kidney PRR was downregulated in different stages of diabetic rats and further suppressed after AT_1_R blockade treatment. Importantly, HRP enhances losartan's therapeutic actions, confirming that PRR activation may be one of the reasons for limited anti-fibrotic effects of AT_1_R blockade on DN and PRR blocker may be a possible new therapy for DN.

## Author contributions

MH and LL contribute to conception and design, data analysis and manuscript writing. LinZ, XA, LiZ, and MH performed animal experiments and data acquisition. XR, DH, and HX performed experiments *in vitro* and analyzed the data. MH and XA performed the histology and immunostaining.

### Conflict of interest statement

The authors declare that the research was conducted in the absence of any commercial or financial relationships that could be construed as a potential conflict of interest.

## References

[B1] Anbar H. S. Shehatou G. S. Suddek G. M. Gameil N. M. (2016). Comparison of the effects of levocetirizine and losartan on diabetic nephropathy and vascular dysfunction in streptozotocin-induced diabetic rats. Eur. J. Pharmacol. 780, 82–92. 10.1016/j.ejphar.2016.03.035 27012991

[B2] Brenner B. M. Cooper M. E. de Zeeuw D. Keane W. F. Mitch W. E. Parving H. H.. (2001). Effects of losartan on renal and cardiovascular outcomes in patients with type 2 diabetes and nephropathy. N. Engl. J. Med. 345, 861–869. 10.1056/NEJMoa011161 11565518

[B3] Deinum J. Ronn B. Mathiesen E. Derkx F. H. Hop W. C. Schalekamp M. A. (1999). Increase in serum prorenin precedes onset of microalbuminuria in patients with insulin-dependent diabetes mellitus. Diabetologia 42, 1006–1010. 10.1007/s001250051260 10491762

[B4] Deng H. X. Shi Y. Furukawa Y. Zhai H. Fu R. Liu E.. (2006). Conversion to the amyotrophic lateral sclerosis phenotype is associated with intermolecular linked insoluble aggregates of SOD1 in mitochondria. Proc. Natl. Acad. Sci. U.S.A. 103, 7142–7147. 10.1073/pnas.0602046103 16636275 PMC1447523

[B5] Ding D. Du Y. Qiu Z. Yan S. Chen F. Wang M.. (2016). Vaccination against type 1 angiotensin receptor prevents streptozotocin-induced diabetic nephropathy. J. Mol. Med. 94, 207–218. 10.1007/s00109-015-1343-6 26407577 PMC4762923

[B6] Gomez R. A. Chevalier R. L. Everett A. D. Elwood J. P. Peach M. J. Lynch K. R.. (1990). Recruitment of renin gene-expressing cells in adult rat kidneys. Am. J. Physiol. 259, F660–F665. 2221104 10.1152/ajprenal.1990.259.4.F660

[B7] He M. Wang Q. Y. Yin Q. Q. Tang J. Lu Y. Zhou C. X.. (2013). HIF-1alpha downregulates miR-17/20a directly targeting p21 and STAT3: a role in myeloid leukemic cell differentiation. Cell Death Differ. 20, 408–418. 10.1038/cdd.2012.130 23059786 PMC3569981

[B8] He M. Zhang L. Shao Y. Wang X. Huang Y. Yao T.. (2009). Inhibition of renin/prorenin receptor attenuated mesangial cell proliferation and reduced associated fibrotic factor release. Eur. J. Pharmacol. 606, 155–161. 10.1016/j.ejphar.2008.12.050 19174157

[B9] He M. Zhang L. Shao Y. Xue H. Zhou L. Wang X. F.. (2010). Angiotensin II type 2 receptor mediated angiotensin II and high glucose induced decrease in renal prorenin/renin receptor expression. Mol. Cell. Endocrinol. 315, 188–194. 10.1016/j.mce.2009.10.008 19879325

[B10] Huang J. Matavelli L. C. Siragy H. M. (2011). Renal (pro)renin receptor contributes to development of diabetic kidney disease through transforming growth factor-beta1-connective tissue growth factor signalling cascade. Clin. Exp. Pharmacol. Physiol. 38, 215–221. 10.1111/j.1440-1681.2011.05486.x 21265872 PMC3077929

[B11] Ichihara A. Hayashi M. Kaneshiro Y. Suzuki F. Nakagawa T. Tada Y.. (2004). Inhibition of diabetic nephropathy by a decoy peptide corresponding to the “handle” region for nonproteolytic activation of prorenin. J. Clin. Invest. 114, 1128–1135. 10.1172/JCI21398 15489960 PMC522242

[B12] Ichihara A. Suzuki F. Nakagawa T. Kaneshiro Y. Takemitsu T. Sakoda M.. (2006). Prorenin receptor blockade inhibits development of glomerulosclerosis in diabetic angiotensin II type 1a receptor-deficient mice. J. Am. Soc. Nephrol. 17, 1950–1961. 10.1681/ASN.2006010029 16738017

[B13] Kaneshiro Y. Ichihara A. Sakoda M. Takemitsu T. Nabi A. H. Uddin M. N.. (2007). Slowly progressive, angiotensin II-independent glomerulosclerosis in human (pro)renin receptor-transgenic rats. J. Am. Soc. Nephrol. 18, 1789–1795. 10.1681/ASN.2006091062 17494887

[B14] Konoshita T. Wakahara S. Mizuno S. Motomura M. Aoyama C. Makino Y.. (2006). Tissue gene expression of renin-angiotensin system in human type 2 diabetic nephropathy. Diabetes Care 29, 848–852. 10.2337/diacare.29.04.06.dc05-1873 16567826

[B15] Lewis E. J. Hunsicker L. G. Bain R. P. Rohde R. D. (1993). The effect of angiotensin-converting-enzyme inhibition on diabetic nephropathy. The collaborative study group. N. Engl. J. Med. 329, 1456–1462. 10.1056/NEJM199311113292004 8413456

[B16] Murali B. Goyal R. K. (2001). Effect of chronic treatment with losartan on streptozotocin induced diabetic nephropathy. Clin. Exp. Hypertens. 23, 513–520. 10.1081/CEH-100106822 11710753

[B17] Nguyen G. Delarue F. Burckle C. Bouzhir L. Giller T. Sraer J. D. (2002). Pivotal role of the renin/prorenin receptor in angiotensin II production and cellular responses to renin. J. Clin. Invest. 109, 1417–1427. 10.1172/JCI0214276 12045255 PMC150992

[B18] Oshima Y. Morimoto S. Ichihara A. (2014). Roles of the (pro)renin receptor in the kidney. World J. Nephrol. 3, 302–307. 10.5527/wjn.v3.i4.302 25374826 PMC4220365

[B19] Price D. A. Porter L. E. Gordon M. Fisher N. D. De'Oliveira J. M. Laffel L. M.. (1999). The paradox of the low-renin state in diabetic nephropathy. J. Am. Soc. Nephrol. 10, 2382–2391. 10541298 10.1681/ASN.V10112382

[B20] Rahimi Z. (2016). The role of renin angiotensin aldosterone system genes in diabetic nephropathy. Can. J. Diabetes 40, 178–183. 10.1016/j.jcjd.2015.08.016 26619914

[B21] Reidy K. Kang H. M. Hostetter T. Susztak K. (2014). Molecular mechanisms of diabetic kidney disease. J. Clin. Invest. 124, 2333–2340. 10.1172/JCI72271 24892707 PMC4089448

[B22] Schefe J. H. Menk M. Reinemund J. Effertz K. Hobbs R. M. Pandolfi P. P.. (2006). A novel signal transduction cascade involving direct physical interaction of the renin/prorenin receptor with the transcription factor promyelocytic zinc finger protein. Circ. Res. 99, 1355–1366. 10.1161/01.RES.0000251700.00994.0d 17082479

[B23] Senbonmatsu T. Saito T. Landon E. J. Watanabe O. Price E. Jr. Roberts R. L.. (2003). A novel angiotensin II type 2 receptor signaling pathway: possible role in cardiac hypertrophy. EMBO J. 22, 6471–6482. 10.1093/emboj/cdg637 14657020 PMC291832

[B24] Siragy H. M. Huang J. (2008). Renal (pro)renin receptor upregulation in diabetic rats through enhanced angiotensin AT1 receptor and NADPH oxidase activity. Exp. Physiol. 93, 709–714. 10.1113/expphysiol.2007.040550 18192338 PMC2586037

[B25] Suzuki F. Hayakawa M. Nakagawa T. Nasir U. M. Ebihara A. Iwasawa A. (2003). Human prorenin has “gate and handle” regions for its non-proteolytic activation. J. Biol. Chem. 278, 22217–22222. 10.1074/jbc.M302579200 12684512

[B26] Tesch G. H. Allen T. J. (2007). Rodent models of streptozotocin-induced diabetic nephropathy. Nephrology 12, 261–266. 10.1111/j.1440-1797.2007.00796.x 17498121

[B27] Zain M. Awan F. R. (2014). Renin Angiotensin Aldosterone System (RAAS): its biology and drug targets for treating diabetic nephropathy. Pak. J. Pharm. Sci. 27, 1379–1391. 25176370

[B28] Zhang J. Gu C. Noble N. A. Border W. A. Huang Y. (2011). Combining angiotensin II blockade and renin receptor inhibition results in enhanced antifibrotic effect in experimental nephritis. Am. J. Physiol. Renal Physiol. 301, F723–732. 10.1152/ajprenal.00271.2011 21795644 PMC3191801

[B29] Zhang L. An X. Wang Q. He M. (2016). Activation of cold-sensitive channels TRPM8 and TRPA1 inhibits the proliferative airway smooth muscle cell phenotype. Lung 194, 595–603. 10.1007/s00408-016-9901-4 27236325

