# Supplementary material for: Inhibition of (pro)renin Receptor Contributes to Renoprotective Effects of Angiotensin II Type 1 Receptor Blockade in Diabetic Nephropathy
Source: Front Physiol. 2017 Oct 6;8:758. doi: 10.3389/fphys.2017.00758 (PMC5635681; doi:10.3389/fphys.2017.00758)
Supplement: Supplementary file 1 [file Table1.DOCX]

Inhibition of the (pro)renin receptor for amelioration of diabetic nephropathy.

Lin Zhang^1^, Xiao-Fei An^4^, Lu-Lu Wo^2^, Xin Ruan^2^, Dong-Dong Huang^2^, Qian Zhao^2^, Li-Min Lu^3^, Ming He^2*^

*^1^Department of Biochemistry and Molecular Cell Biology; ^2^Key Laboratory of Cell Differentiation and Apoptosis of Chinese Ministry of Education, Department of Pathophysiology, Shanghai Jiao Tong University School of Medicine (SJTU-SM), Shanghai, China. ^3^Department of Physiology and Pathophysiology, Fudan University Shanghai Medical College, Shanghai, China; ^4^Department of Endocrinology, Jiangsu Province Hospital of Chinese Medicine, Affiliated Hospital of Nanjing University of Chinese Medicine, Nanjing, Jiangsu, China.*

*Corresponding author. Ming He, Key Laboratory of Cell Differentiation and Apoptosis of Chinese Ministry of Education, Department of Pathophysiology, Shanghai Jiaotong University School of Medicine (SJTU-SM), 280 Chongqing South Road, Shanghai 200025, China. Tel.: +86-21-63846590-776403. E-mail: [heming@shsmu.edu.cn](mailto:heming@shsmu.edu.cn).

**Table S1** Sequences and accession numbers for primers of rat used in real-time PCR.

| Gene | Primer | Sequence (5′-3′) | Accession No. |
| --- | --- | --- | --- |
| PRR | Forward | CATAAGCATCTCGCCAAGG | AB188298 |
|  | Reverse | ACCAGGGATGTGTCGAATGA |  |
| AT_1_R | Forward | CTCAAGCCTGTCTACGAAAATGAG | M86912 |
|  | Reverse | TAGATCCTGAGGCAGGGTGAAT |  |
| AT_2_R | Forward | ACCTTTTGAACATGGTTTG | U01908 |
|  | Reverse | GTTTCTCTGGGTCTGTTTGCTC |  |
| (pro)renin | Forward | GATCACCATGAAGGGGGTCTCTGT | NM_012642 |
|  | Reverse | GTTCCTGAAGGGATTCTTTTGCAC |  |
| TGF-β1 | Forward | TGGCGTTACCTTGGTAACC | NM_021578 |
|  | Reverse | GGTGTTGAGCCCTTTCCAG |  |
| GAPDH | Forward | CCTTCATTGACCTCAACTACATG | NM_017008 |
|  | Reverse | CTTCTCCATGGTGGTGAAGAC |  |

PRR, (pro)renin receptor; AT_1_R, Angiotensin II type 1 receptor; AT_2_R, Angiotensin II type 2 receptor;TGF, transforming growth factor.
